# Immune dysregulation is an important factor in the underlying complications in Influenza infection. ApoH, IL-8 and IL-15 as markers of prognosis

**DOI:** 10.3389/fimmu.2024.1443096

**Published:** 2024-07-26

**Authors:** Sara Garcinuño, Antonio Lalueza, Francisco Javier Gil-Etayo, Raquel Díaz-Simón, Ignacio Lizasoain, Ana Moraga, Blanca Diaz-Benito, Laura Naranjo, Oscar Cabrera-Marante, Daniel Enrique Pleguezuelo, Maria Ruiz-Ruigomez, Blanca Ayuso, Estibaliz Arrieta, Dolores Folgueira, Estela Paz-Artal, Cecilia Cueto, Carlos Lumbreras, Antonio Serrano, Manuel Serrano

**Affiliations:** ^1^ Healthcare Research Institute Hospital 12 de Octubre (imas12), Hospital Universitario 12 de Octubre, Madrid, Spain; ^2^ Immunology Department, Hospital Universitario 12 de Octubre, Madrid, Spain; ^3^ Internal Medicine Department, Hospital Universitario 12 de Octubre, Madrid, Spain; ^4^ Faculty of Medicine, Universidad Complutense, Madrid, Spain; ^5^ Red de Infecciones en Inmunodeprimidos no VIH e infecciones relacionadas con la asistencia sanitaria (CIBERINFEC), Instituto de Salud Carlos III, Madrid, Spain; ^6^ Cell Biology Department, Faculty of Medicine, Universidad Complutense, Madrid, Spain; ^7^ Microbiology Department, Hospital Universitario 12 de Octubre, Madrid, Spain; ^8^ Biochemistry Department, Hospital Universitario 12 de Octubre, Madrid, Spain; ^9^ Red de Centros de Investigación Biomédica en Epidemiología y Salud Pública (CIBERESP), Instituto de Salud Carlos III, Madrid, Spain

**Keywords:** influenza, flu, apolipoprotein H, ApoH, β2GPI, IL15, IL8, early immune response

## Abstract

**Introduction:**

Influenza virus infection can cause a range of clinical symptoms, including respiratory failure (RF) and even death. The mechanisms responsible for the most severe forms of the disease are not yet well understood. The objective is to assess the initial immune response upon admission and its potential impact on infection progression.

**Methods:**

We conducted a prospective observational study of patients with influenza virus infection who required admission to a tertiary hospital in the 2017/18 and 2018/19 flu seasons. Immune markers, surrogate markers of neutrophil activation, and blood levels of DNase I and Apolipoprotein-H (ApoH) were determined in the first serum sample available during hospital care. Patients were followed until hospital discharge or death. Initially, 792 patients were included. From this group, 107 patients with poor evolution were selected, and a random control group was matched by day of admission.

**Results:**

Patients with poor outcomes had significantly reduced ApoH levels, a soluble protein that regulate both complement and coagulation pathways. In multivariate analysis, low plasma levels of ApoH (OR:5.43; 2.21-13.4), high levels of C- reactive protein (OR:2.73: 1.28-5.4), hyperferritinemia (OR:2.83; 1.28-5.4) and smoking (OR:3.41; 1.04-11.16), were significantly associated with a worse prognosis. RF was independently associated with low levels of ApoH (OR: 5.12; 2.02-1.94), while high levels of IL15 behaved as a protective factor (OR:0.30; 0.12-0.71).

**Discussion:**

Therefore, in hospitalized influenza patients, a dysregulated early immune response is associated with a worse outcome. Adequate plasma levels of ApoH are protective against severe influenza and RF and High levels of IL15 protect against RF.

## 1 Introduction

Influenza is a contagious disease due to influenza viruses characterized by involvement of the respiratory tract that appears in seasonal epidemics and annually affect between 8 and 10% of the population (1, 2). Clinical manifestations present a wide spectrum of clinical profiles, from a self-limited mild infection to complications such as severe pneumonia, respiratory failure (RF), multiple organ failure or even death, reported in 20% of serious infections (3, 4). The mechanisms and factors associated with poor outcomes are not yet well understood.

Variables associated with complications have been identified that could serve as possible prognostic factors, including age, delay in the administration of antiviral treatment, pneumonia, thrombocytopenia, lymphopenia and PaO2/FiO2<200 mmHg (5–8).

As has already been reported in other infections, the evolution of the pathology based on the immune response established around the infection by influenza virus could be a determining factor in the evolution of patients (9–11). The secretion of cytokines by respiratory epithelial cells during infection is a determining factor in the disease's prognosis. An imbalance of cytokines can lead to ineffective and exaggerated adaptive immune responses (12–14), which could result in multi-organ failure and lung tissue damage (15).

Neutrophil extracellular traps (NETs) are a form of immediate response against any type of infection that consists of the release of DNA and molecules from the neutrophil cytoplasm, mainly antimicrobial peptides and proteases, which are organized in a network where pathogens are trapped and destroyed (16). Although NETs can prevent the spread of the infectious disease, their release and degradation by DNases after infection must be tightly regulated to effectively control the infection but avoiding excessive inflammatory reactions (17, 18). High levels of NETs causes tissue damage due to the activity of enzymes associated with NETs, such as mieloperoxidase (MPO) and elastases and correlates with poor prognosis of severe Influenza A infection (19). Dysregulation of the NETs formation process has been associated with the initiation and progression of autoimmune and autoinflammatory diseases (20). Various soluble proteins of the innate immune system such as C-reactive protein (CRP), ferritin, DNase-I and apolipoprotein H (ApoH) could play an important role, both systemically and locally, in the early stages of infection, to contain it, until a specific response is developed (21–23). During this early immune response, there is an increase in hepatic synthesis and in the plasmatic concentration of a group of proteins, which are involved in the inflammatory process as immune regulators (24, 25).

ApoH, also known as Beta 2 Glycoprotein 1 (β2GPI), is a soluble protein that regulates both complement and coagulation (26–28). ApoH also plays an important role as an opsonin: it can bind to microvesicles containing phosphatidylserine, such as microorganisms, viruses, vesicles or apoptotic bodies with proinflammatory activity (29). ApoH mediates virus and debris clearance (30) and is especially known to be the main antigenic target of antiphospholipid antibodies, present in patients with antiphospholipid syndrome (28).

The activity of the molecules involved in early immune responses must be strictly regulated to guarantee infection control and avoid triggering excessive inflammatory reactions that can lead to multiple organ failure or favour the appearance of numerous pathologies such as systemic lupus erythematosus (SLE) (17, 31, 32). In the present work we study the presence of the main plasma markers related to the innate immune response in the first moments of influenza infection and their relationship with patient's evolution.

## 2 Materials and methods

### 2.1 Study design

A prospective observational study enrolled adults (>18 years) with confirmed influenza at Hospital 12 de Octubre (Madrid, Spain) during 2017/18 and 2018/19 seasons (December to March in both periods). For inclusion, it was mandatory that a serum sample drawn within 24 hours of admission was available. Patients were followed up until hospital discharge or death. Demographic and clinical data were collected from electronic records. Collection of demographic and clinical laboratory data was obtained from the electronic medical record. Laboratory parameters included the absolute number of leukocytes, lymphocytes, platelets, C-reactive protein (CRP) and ferritin. Admission occurred with a median of 2 days after symptom manifestation.

### 2.2 Patients

From a total of 792 patients that were recruited, 107 had a poor evolution (see definition below) and the rest had an uneventful evolution until discharge.

Due to the impossibility of evaluating the early immune response in all patients, a two-cohort study was carried out. In this way, it was possible to study all the patients with poor evolution and compare them with a similar number of patients with good evolution.

**Figure 1:**
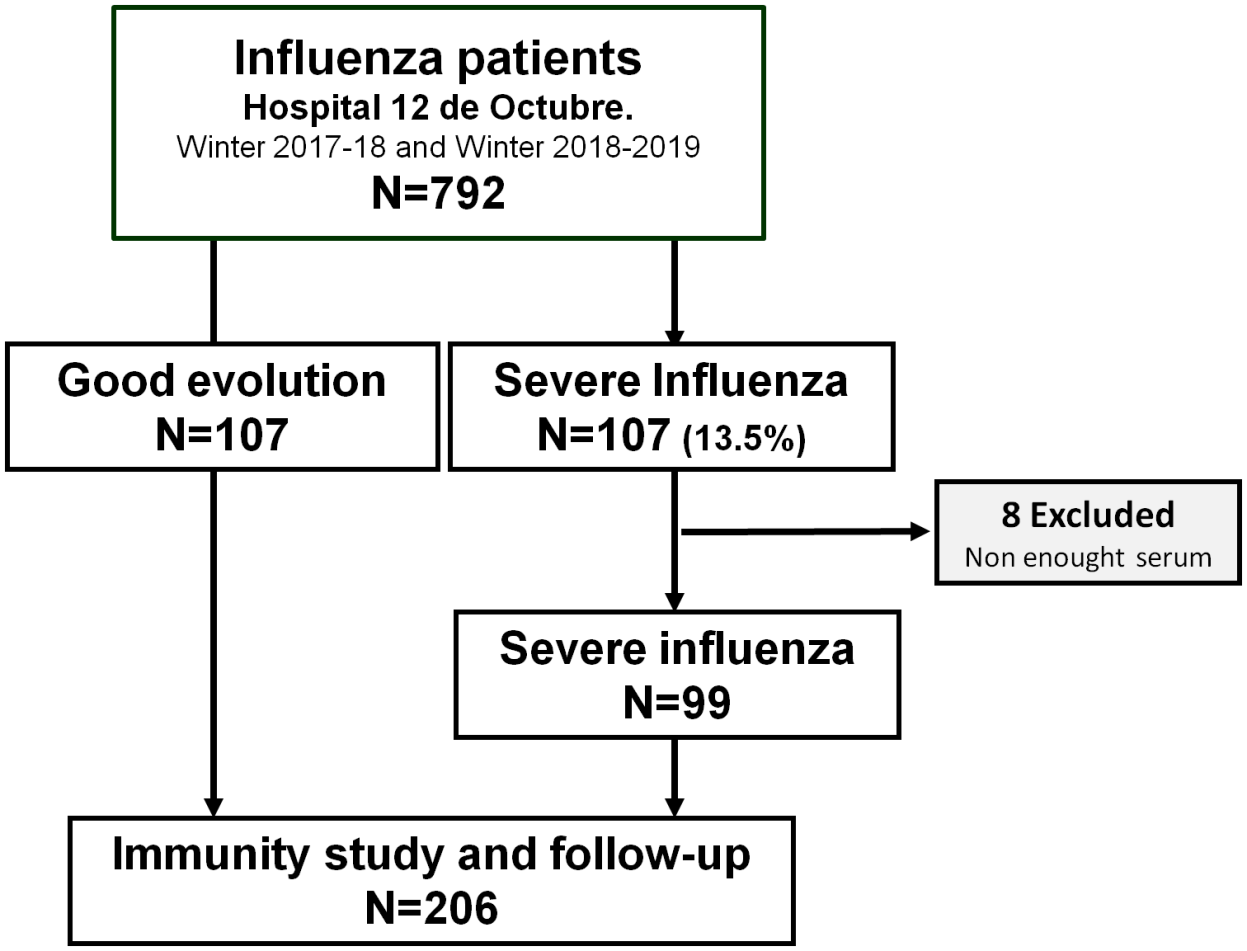
Algorithm of the distribution of patients. From the total group of 792 patients, 107 presented poor evolution. For each patient with severe influenza, a control was randomly selected from among the mild patients group.

In the first cohort, all patients with severe complications were incorporated. The second cohort was formed by a control group built with 107 patients with good evolution. For each patient with severe flu, a control was randomly selected from among the mild patients with sample availability who were admitted on the same day as the severe patient. In 8 of the 107 patients with poor evolution the serum sample was insufficient to be able to carry out an adequate immunological study, so they were discarded although their corresponding control remained in the study. Finally, 206 patients with influenza were part of the study, 99 with poor evolution and 107 with good evolution. See arrangement and algorithm in **Figure 1**.

In addition, a reference group made up of 41 anonymous blood donors was created to compare the profile of cytokines and other factors of innate immunity between healthy people and patients. This control group included healthy people who went to our hospital to donate blood. All of them tested negative for influenza virus and signed a questionnaire declaring that they did not suffer from any disease.

### 2.3 Study definitions

Severe Influenza (bad evolution) included RF, Intensive Care Unit (ICU) admission, or death during hospitalization. Good evolution was defined as the absence of any of these complications.

Respiratory failure (RF) was defined as PaO2/FiO2 ratio <200 mmHg or the need for mechanical ventilation, including those patients who had a clinical indication for ventilatory support but for any reason were finally not ventilated. If PaO2 was unavailable, estimated PaO2/FiO2 ratio was calculated using SpO2/FiO2 ratio (33).

The cut-off points used for the haematological abnormalities were as follows:

Leukocytosis (leukocytes >11,000/µL), leukopenia (leukocytes <4,000/µL), lymphopenia (lymphocytes <1,000/µL), severe lymphopenia (<500/µL), and thrombocytopenia (platelet <140,000/µL).

Obesity was defined as a Body Mass Index (BMI) >30.

### 2.4 Samples

Serum samples were collected and processed during the first 24 hours after admission to the emergency department. Admission occurred with a median of 2 days after the manifestation of clinical symptoms, without significant differences between patients with good or poor evolution (p=0.803).

### 2.5 Immune parameters

Cytokine determination assays Serum cytokine profile, including IFNα, IFNγ, IL-2, IL-6, IL-10, and IL-15, was determined by the Human Cytokine Magnetic Beads Panel Kit (EMD Millipore Corporation, St. Charles, MO, 63304, USA) using a LABScan ™ 100 Luminex. The results were analyzed with the Luminex xPONENT42 v3.1 software. In addition, serum IL-8 was assessed by Flex ELISA: Human IL-8 CXCL8 (Mabtech AB, Nacka Strand, Sweden). The experimental procedure was carried out following the manufacturer's recommendations and automated in a Triturus^®^ Analyzer (Diagnostics Grifols SA, Barcelona, Spain).

Consideration of elevated or decreased immune-markers levels (cytokines, DNase, ApoH and MPO) for dichotomization and use as potential biomarkers was performed using the mean ± 2 standard deviation (STD) of each marker in a group of 41 healthy blood donors as the cutoff. The cutoff points for cytokines are described in **Supplementary Table S1**).

ApoH determination assay Serum β2GPI levels were quantified using ELISA Pro Human ApoH (Mabtech AB, following the manufacturer's instructions. The ELISA procedures were carried out in a Triturus^®^ Analyzer (Diagnostics Grifols SA). Low levels of β2GPI were considered to be values ≤86 mg/l, corresponding to the mean levels previously described in healthy people (178 ± 46 mg/l) minus twice the STD (34).

### 2.6 DNase activity assay

Serum DNase activity was measured with a functional technique that assesses DNA hydrolysis in a semisolid medium. The plates were prepared with PBS Buffer (with Ca and Mg, pH 7.4) to which 10 mg/mL of agarose, 0.13 mg/mL of DNA and 0.2 mg/mL Syber Green (Invitrogen, Carlsbad, California, USA) were added. Small cylindrical holes (2 mm diameter) were created in which 10uL of the serum sample were placed and incubated for 16h at 37°C. The halos resulting from DNA hydrolysis were evaluated in a Syngene™ LED Blue Light transilluminator (Syngene, Bangalore, India). DNase levels were calculated by relating them to the activity of pure DNase (Manufacturer Invitrogen, Carlsbad, California, USA) diluted to known concentrations. Serum DNase activity levels ≤ 12 ng/ml were considered low.

### 2.7 MPO determination assay

Human myeloperoxidase (MPO) is the main biocidal protein in NETs (35). For this reason, we determined levels of MPO in serum as an indirect measurement of NETosis activity using Human MPO Quantikine ELISA kit (R&D Systems, Minneapolis, MN, USA). The procedure was performed following the manufacturer's instructions. Briefly, samples were diluted 50-fold in assay diluent and incubated for 2 hours at room temperature. Subsequently, the samples were then washed, the conjugate was added to the plate and incubated for 2 hours. After new washes, substrate solution was added and incubated for 30 minutes. Samples were read at 450 nm. Elevated levels of MPO in the blood were considered to be values > 1783 ng/mL (mean value + 2 STD observed in a sample of 41 blood donors).

### 2.8 Microbiological methods

For influenza molecular diagnosis, the Allplex^™^ respiratory Panel 1 (Seegene, Seoul, South Korea) was utilized. This panel enables the simultaneous detection of Flu A, Flu B, RSV A, RSV B and Flu A subtypes H1, H1pdm09 and H3. Nucleic acid extraction (200 μl assay volume) was carried out using the MicrolabStarlet IVD with the STARMag 96 x 4 Universal Cartridge Kit (Seegene, Seoul, South Korea). The rRT-PCR, was performed on the CFX96^™^ system (Bio-Rad Laboratories, Hercules, CA, USA). Analysis of the results was done using Seegene viewer software.

### 2.9 Ethics statement

This study was carried out in accordance with the principles of the Declaration of Helsinki and was approved by the Clinical Research Ethics Committee of Hospital Universitario 12 de Octubre (reference numbers 20/117, 18/182 and 18/009). The patients signed informed consent.

### 2.10 Statistical analysis

The results of the scaled variables were expressed as median with interquartile range (IQR) in brackets. For comparisons between two groups, the Wilcoxon-Mann-Whitney test was used. To compare the variability in the continuous variables between cohorts, the Hodges-Lehmann median difference and the relative difference of the Hodges Lehman medians (RMD) were used. The RMD was calculated by dividing the Hodges Lehman median difference by the highest value of the cohort medians. This index varies between 0 and 1, with the values closest to zero indicating very small (irrelevant) differences. When more than two groups were compared, the Kruskal–Wallis test was used.

The results of the qualitative variables were expressed in absolute frequency and percentage. The association between variables was determined using the Chi-square test or Fisher's exact test, when appropriate. The relative size of an effect is expressed as an odds ratio (OR). Multivariate analyzes were performed using a logistic regression model. Data were analyzed with MedCalc for Windows version 20.1 (MedCalc Software, Ostend, Belgium), Microsoft Excel (Microsoft Corp, Redmond, WA, USA), and GraphPad Prism version 9 (GraphPad Software,Boston, MA, USA). Probabilities less than 0.05 were considered significant.

## 3 Results

### 3.1 Characteristics of the patients and controls

Analysing the whole group of influenza patients, the median age was 75.5 (IQR: 62-85) years with a balanced distribution by gender (women 54.4%). The most prevalent comorbidities were ex-smoking habit (25.2%), Diabetes Mellitus (23.8%) and obesity (22.3%) (**Supplementary Table S2**). In 94% of cases, the infection was caused by influenza A, while influenza B accounted for the remaining 6%. Among cases of influenza A, the most common subtype was H3N2 (79%), with the remaining 21% caused by subtype H1N1pmd09.

Comparing all influenza patients with healthy population (**Supplementary Table S3**), influenza patients presented a lower concentration of alpha and gamma interferons (IFN α: 1.3 vs 7.0 pg/mL p<0.001; IFNγ: 0.5 vs 4.8 pg/mL, p=0.002) and a higher concentration of IL-10 (10.8 vs 0.9 pg/mL, p<0.001) and IL-15 (2.3 vs 0.2 pg/mL, p<0.001). MPO levels were higher in patients with influenza: 1154 (486-2379) vs 396 (266-708) ng/ml, p<0.001.

Influenza patients presented a decreased DNase activity compared to healthy controls: 8 (4-8) vs 108 (77-138) ng/mL, p<0.001 and a slightly lower ApoH concentration although the differences were not significant: 177 (103-281) vs 209 (168-256) ug/mL, p=0.079 (**Supplementary Table S3**).

### 3.2 Patients with influenza according to the severity of the disease

The comparison of the demographic data of patients with severe influenza with those who had a favourable evolution showed that patients with severe influenza were younger: 71 (57-83) vs 80 (68-86) years old (p=0.004) having also a higher smoking prevalence (16.2% vs 4.7%. p=0.007) and a lower obesity proportion, (15.2% vs 28.9%. p=0.017). Additionally, severe patients showed a higher number of leukocytes per µL: 9500 (6900-13100) vs 7700 (5825-10900; p=0.012), CRP: 13 (5.3-26.25) vs 7 (3-13) µg/mL (p<0.001), ferritin: 452 (186-959) vs 235 (149-428), ng/mL (p<0.001), MPO: 1571 (673-2884) vs. 1038 (473-1733) ng/ml (p=0.017) and IL-8: 22 (3.5-61.5) vs 3.5 (3.5-24.4), pg/mL, (p= 0.001) (**Table 1**).

**Table 1:** Comparison of the input parameters of patients with influenza who had a poor outcome versus those who had a course without serious complications.

	BAD EVOLUTION N=99		GOOD EVOLUTION N=107		p-value	OR	95% CI
CONDITION	N/median	IQR/%	N/median	IQR/%			
Sex (women)	54	(54.5%)	58	(54.2%)	0.961		
Age (years)	71	(57-83)	80	(68-86)	0.004		
BMI	25	(23-31)	29	(26-32.5)	0.009		
Days hospitalized	12	(9-21)	6	(4-9.75)	< 0.001		
IFNa (pg/mL)	1.31	(1.1-1.6)	1.6	(1.3-2.2)	0.107		
IFNg (pg/mL)	0.4	(0.4-3.6)	0.5	(0.5-5.7)	0.075		
IL-8 (pg/mL)	22	(3.5-61.5)	3.5	(3.5-24.4)	0.001		
IL-10 (pg/mL)	12.96	(5.06-43)	8.3	(0.9-19)	0.022		
IL-15 (pg/mL)	3.09	(0.3-7.3)	0.3	(0.23-8.8)	0.002		
IL-2 (pg/mL)	0.02	(0.01-0.1)	0.1	(0.02-0.1)	< 0.001		
IL-6 (pg/mL)	14.5	(3.6-51.5)	9	(2.2-24.1)	0.101		
MPO (pg/mL)	1571	(673-2884)	1038	(473-1733)	0.017		
APOH (ug/mL)	144	(82-230)	205	(130-338)	< 0.001		
DNase (ng/mL)	8	(8-8)	8	(4-8)	0.080		
Leukocytes/uL	9500	(6900-13100)	7700	(5825-10900)	0.012		
Lymphocytes/uL	629	(390-1150)	742	(504-1238)	0.055		
Platelets (*10^3^/uL)	200	(136-269)	201	(158-276)	0.330		
Ferritin (ng/mL)	452	(186-959)	235	(149-428)	< 0.001		
CRP (ug/mL)	13	(5.3-26.3)	7	(3-13)	< 0.001		
Biomarkers with possible association							
Smoker	16	(16.2%)	5	(4.7%)	0.007	3.93	(1.38-11.18)
Former smoker	27	(27.3%)	25	(23.4%)	0.519		
Obesity	15	(15.2%)	31	(28.9%)	0.017	0.44	(0.22-0.87)
COPD	26	(26.3%)	17	(15.9%)	0.067		
Diabetes Mellitus	25	(25.3%)	24	(22.4%)	0.635		
IFNa elevated	9	(9.1%)	13	(9.1%)	1.000		
IFNg elevated	12	(12.1%)	7	(6.7%)	0.180		
IL8 elevated	45	(62.5%)	31	(39.2%)	0.004	2.58	(1.34-4.98)
IL10 elevated	28	(28.3%)	19	(18.1%)	0.084		
IL15 elevated	25	(25.3%)	34	(32.4%)	0.262		
IL2 elevated	1	(1%)	7	(6.7%)	0.038	0.14	(0.02-1.18)
IL6 elevated	18	(18.2%)	11	(10.5%)	0.115		
MPO elevated	28	(43.1%)	19	(23.2%)	0.010	2.51	(1.23-5.1)
ApoH low	30	(30.3%)	9	(8.4%)	< 0.001	4.73	(2.11-10.6)
DNase low	91	(91.9%)	91	(85.8%)	0.169		
CRP elevated	31	(32%)	13	(12.1%)	0.001	3.4	(1.65-6.98)
Ferritin elevated	45	(47.4%)	22	(21%)	< 0.001	3.4	(1.83-6.31)
Leucocytosis	34	(35.1%)	26	(24.3%)	0.092		
Leucopenia	5	(5.2%)	5	(4.7%)	0.874		
Lymphopenia	68	(70.1%)	65	(60.7%)	0.162		
Severe lymphopenia	39	(39.8%)	27	(25.2%)	0.026	1.96	(1.08-3.55)
Thrombopenia	25	(25.5%)	16	(15%)	0.059		

BMI: body mass index; MPO: myeloperoxidase; COPD: chronic obstructive pulmonary disease; CRP: C-reactive protein.

Comparing the cytokine profile between patients with severe form and those with good evolution, it was detected that those with severe form presented an altered immune serum-proteins profile (**Table 1**).

**Figure 2:**
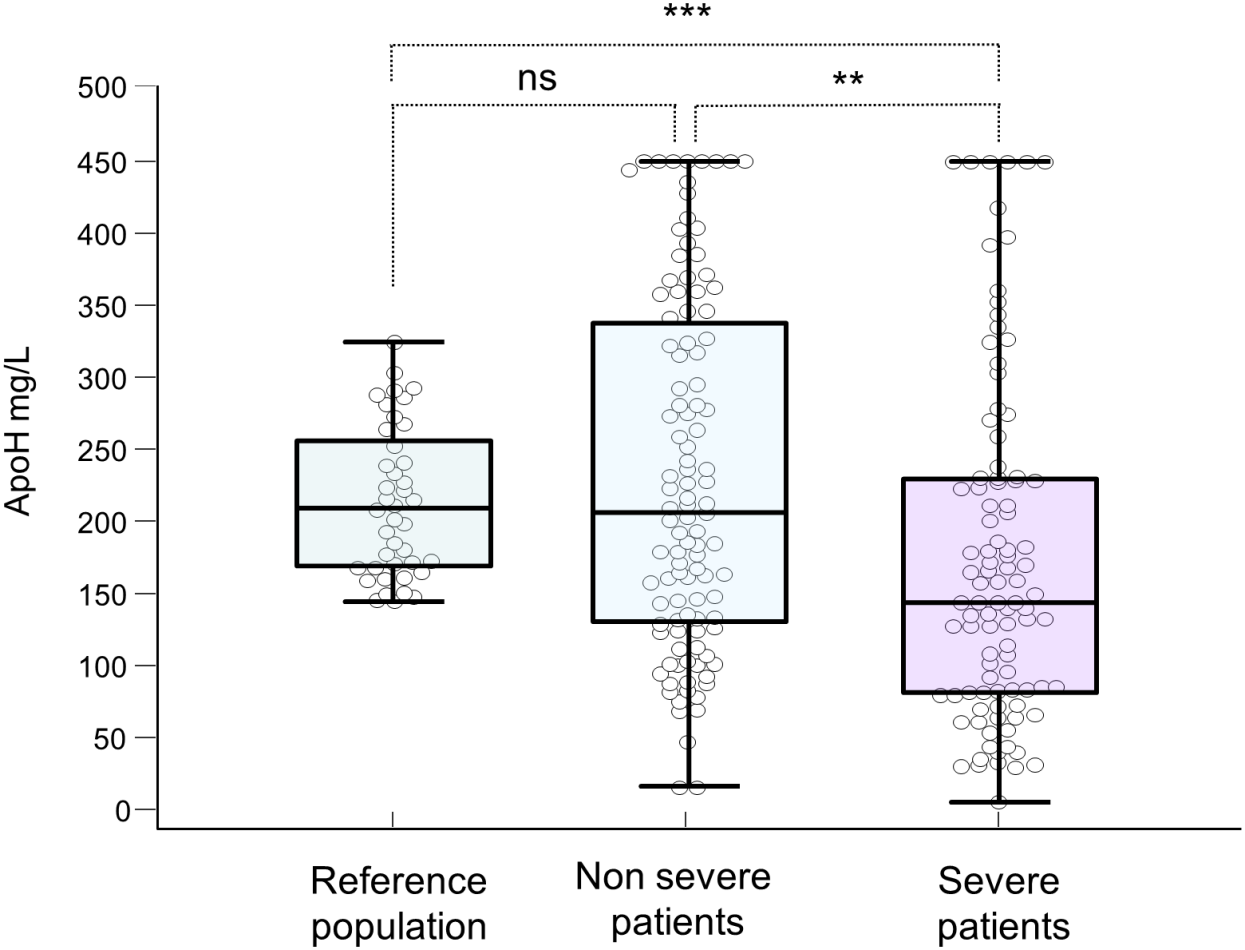
Blood levels of Apolipoprotein H in the blood (mg/L) of healthy people, mild and severe patients. **: p-value ≤ 0.01; ***:p-value ≤ 0.001; ns: not significant. p-value > 0.05.

Severe patients showed a marked reduction of ApoH levels compared to those with good evolution: 144 (82-230) vs 205 (130-338) µg/mL (p<0.01) and compared with healthy controls: 209 (168-256) µg/mL (p=0.001) (**Figure 2**). The proportion of patients with low ApoH levels was higher in patients with severe form than in those with good evolution (30.3% vs 8.4%, p <0.001, **Table 1**). No significant differences were observed in the levels of DNase activity and in the proportion of patients with low DNase between patients with severe or mild forms of the disease.

A first multivariate analysis was performed with those variables associated with poor outcome that had shown a p value < 0.05 in the previous univariate analysis (**Table 2A**), in which, elevated levels of IL8, MPO and ferritin did not behave as independent variables. Since these three variables are associated with the formation of NETs and increase together in its formation (would be interdependent), it was performed a new multivariate analysis in which only one of the variables associated with NETs was assessed: ferritin levels (see discussion below). In this second multivariate analysis, smoking habit (OR 3.41), low levels of ApoH (OR 5.43) and high levels of Ferritin (OR 2.63) and CRP (OR 2.73) behaved as independent risk factors associated with poor outcome (**Table 2B**).

**Table 2:** Multivariate analysis of the markers that were associated with poor flu outcomes detected in a first univariate analysis of influenza patients.

	BAD EVOLUTION		BAD EVOLUTION		
	UNIVARIATE		MULTIVARIATE		
Biomarker	Odds ratio	95% CI	Odds ratio	95% CI	P value
A. All significant variables					
Age (per year)	0.98	(0.96-0.99)	0.97	(0.94-1)	0.054
Smoker	3.93	(1.38-11.18)	Not	evaluable	
Obesity	0.44	(0.22-0.87)	0.17	(0.05-0.66)	0.010
IL8 elevated	2.58	(1.34-4.98)	1.20	(0.43-3.33)	0.723
MPO elevated	2.51	(1.23-5.1)	0.93	(0.32-2.73)	0.894
ApoH low	4.73	(2.11-10.6)	3.98	(1.01-5.66)	0.048
CRP elevated	3.40	(1.65-6.98)	4.26	(1.28-4.14)	0.018
Severe lymphopenia	1.96	(1.08-3.55)	3.99	(1.4-1.32)	0.009
Ferritin elevated	3.40	(1.83-6.31)	1.25	(0.41-3.77)	0.696
Area under ROC curve			0.789	(0.706-0.858)	
B. Excluding IL8 and MPO					
Age (years)	0.98	(0.96-0.99)	0.98	(0.96-1)	0.050
Smoker	3.93	(1.38-11.18)	3.41	(1.04-11.16)	0.043
Obesity	0.44	(0.22-0.87)	0.50	(0.22-1.11)	0.090
ApoH low	4.73	(2.11-10.6)	5.43	(2.21-3.4)	<0.001
CRP elevated	3.40	(1.65-6.98)	2.73	(1.2-6.21)	0.017
Severe lymphopenia	1.96	(1.08-3.55)	1.98	(0.98-4)	0.058
Ferritin elevated	3.40	(1.83-6.31)	2.63	(1.28-5.4)	0.008
Area under ROC curve			0.790	(0.727-0.844)	

MPO: myeloperoxidase; CRP: C-reactive protein.

Besides, although not significant in the multivariate study, there was a higher incidence of the H1N1pmd09 subtype in patients with a worse prognosis (27% vs 13%, p<0.001) (**Supplementary Table S4**).

### 3.3 Patients with respiratory failure

Sixty-one patients suffered RF. Patients with RF were younger than the rest: 70 (57-80) vs 79 (66-86) years, (p<0.001) and had higher rates of smoking (23% vs 4.8%, p<0.001) and COPD (32.8% vs 15.9%, p=0.006). Also had a higher prevalence of high levels of CRP, IL8, IL10 and MPO than in those with normal ventilatory function (OR 2.42, 2.69, 2.36 and 2.33 respectively). Patients with RF presented higher proportion of patients with low ApoH levels (OR: 4.25, p <0.001) (**Table 3**).

**Table 3:** Characteristics of influenza patients with respiratory failure versus those who did not have respiratory complications.

	Respiratory Failure N=61		No Respiratory failure N=145		p-value	OR	95% CI
CONDITION	N/median	IQR/%	N/median	IQR/%			
Sex (women)	33	(54.1%)	79	(54.5%)	0.960	1.01	(0.59-1.75)
Age (years)	70	(57-80)	79	(66-86)	< 0.001		
BMI	26	(24-31)	28	(24.7-32)	0.213		
Days hospitalized	12	(9-21)	6	(4-9.75)	< 0.001		
Treated at ICU	32	(52.5%)	15	(10.3%)	< 0.001	9.56	(4.59-19.92)
Smoker	14	(23%)	7	(4.8%)	< 0.001	5.87	(2.24-15.43)
Former smoker	17	(27.9%)	35	(24.1%)	0.574		
Obesity	10	(16.4%)	36	(24.8%)	0.185		
COPD	20	(32.8%)	23	(15.9%)	0.006	2.59	(1.29-5.19)
Diabetes Mellitus	13	(21.3%)	36	(24.8%)	0.588		
IFNa elevated	2	(3.3%)	2	(1.4%)	0.265		
IFNg elevated	7	(11.5%)	12	(8.4%)	0.488		
IL8 elevated	29	(67.4%)	47	(43.5%)	0.008	2.69	(1.28-5.65)
IL10 elevated	21	(34.4%)	26	(18.2%)	0.012	2.36	(1.2-4.65)
IL15 elevated	11	(18%)	48	(33.6%)	0.025	0.44	(0.21-0.91)
IL2 elevated	0	(0%)	8	(5.6%)	0.055		
IL6 elevated	10	(16.4%)	19	(13.3%)	0.561		
MPO elevated	18	(46.2%)	29	(26.9%)	0.027	2.33	(1.09-4.99)
ApoH low	22	(36.1%)	17	(11.7%)	< 0.001	4.25	(2.05-8.79)
DNase low	55	(90.2%)	127	(88.2%)	0.683		
CRP elevated	20	(32.8%)	24	(16.8%)	0.011	2.42	(1.21-4.83)
Ferritin elevated	25	(42.4%)	42	(29.8%)	0.087		
Leucocytosis	19	(31.7%)	41	(28.5%)	0.648		
Leucopenia	3	(5%)	7	(4.9%)	0.273		
Lymphopenia	44	(73.3%)	89	(61.8%)	0.116		
Severe lymphopenia	23	(37.7%)	43	(29.9%)	0.272		
Thrombopenia	15	(36.6%)	46	(28%)	0.285		

BMI, body mass index; ICU, Intensive Care Unit; COPD, chronic obstructive pulmonary disease; MPO, myeloperoxidase; CRP, C-reactive protein.

A first multivariate analysis was performed including the variables that in a previous univariate analysis were significant. Four variables were identified as independent, all with statistical significance: smoking, COPD and low ApoH, behaved as independent risk factors and high levels of IL15 behaved as a protective factor (**Supplementary Table S5**). As eight variables were analysed over 61 events, with a ratio of only 7.6 events per variable (in the literature 10 are recommended) (36), a second analysis was carried out, restricting to six variables. The variables smoking, low levels of ApoH, high levels of CRP and high levels of IL10 behaved as independent and significant risk factors (OR: 5.25, 5.12, 2.97 and 3.12, p<0.01) while high levels of IL15 behaved as an independent protective factor (OR: 0.30, p<0.01) (area under the ROC curve 0.788, 95%CI: 0.725-0.842) (**Table 4**).

**Table 4:** Multivariate analysis of the markers associated with respiratory failure in the 61 influenza patients who had this evolution.

	Respiratory failure		Respiratory failure		
	UNIVARIATE		MULTIVARIATE		
Biomarker	Odds ratio	95% CI	Odds ratio	95% CI	P value
Smoker	5.87	(2.24-15.43)	5.25	(1.57-17.51)	0.007
COPD	2.59	(1.29-5.19)	2.24	(0.93-5.39)	0.073
IL10 elevated	2.36	(1.2-4.65)	3.12	(1.39-6.98)	0.006
IL15 elevated	0.44	(0.21-0.91)	0.30	(0.12-0.71)	0.007
ApoH low	4.25	(2.05-8.79)	5.12	(2.02-1.94)	<0.001
CRP elevated	2.42	(1.21-4.83)	2.97	(1.33-6.64)	0.008
Area under ROC curve			0.788	(0.725-0.842)	

COPD, chronic obstructive pulmonary disease; CRP, C-reactive protein.

## 4 Discussion

Our work would suggest that the initial state of innate immunity during influenza infection may have a significant impact on clinical progression. It is noteworthy that in our study, patients with severe forms were younger and less obese than those who suffered a less severe disease. These results contrast with those who reported that age could be related to disease severity (37, 38). This discrepancy may arise from potential biases in the hospital admission of older or fatter people, who are admitted even if they suffer from very mild forms because they have a greater risk of suffering complications, in order to be able to act quickly if problems arise. These results could suggest that elderly, who are supposed to present more complications, are well monitored and controlled seasonally. In this study, the decision on the need for hospital admission was made by the Emergency Department physicians before the patient was assessed and included by the research team.

CRP stands out as independent risk factor for disease severity aligning with previous findings in both influenza and SARS-CoV-2 (34, 39, 40). In the same way high levels of ferritin have been identified as an independent risk factor for disease severity, as previously reported. The identification of high ferritin levels as an independent risk factor for severe influenza is consistent with previous studies (41). Ferritin is a key regulator in iron homeostasis and it is considered an acute inflammatory reactant. Nowadays it is suggested that ferritin is an important modulator of the innate and adaptive immune system (42–44).

Severe patients presented significantly higher levels of IL-8 as previously reported (15). The measurement of IL-8 during the acute phase proves crucial, as it correlates strongly with clinical complications. IL-8 is a potent chemoattractant and activator for myeloid leukocytes and neutrophils which acts through CXCR1, CXCR2 and NF-κB (15, 45). IL-8 is implicated in neutrophils' recruitment to infectious or tumorigenic areas, promoting either protective or harmful immune responses (45). During respiratory viral infections, epithelial respiratory cells secrete a vast number of cytokines including IL-8 (46).

One of the main causes of NETosis is the potent activation of neutrophils via IL-8. Inside their granules exists a weaponry of antimicrobial molecules. Bacterial, viral or fungal infection are able to activate neutrophils, favouring the secretion of NETs (16). Although NETs are beneficial for the clearance of infections, they have been related to pathological conditions like pulmonary diseases, immunothrombosis or autoimmunity (47).

Recently it has been described that ferritin induces the formation of NETs in a way mediated by Macrophage Scavenger Receptor 1 (Msr1). Ferritin acts as a MSr1 ligand and trigger the NET formation pathway (48). This mechanism explains why in our study the biomarkers associated with NET activation (MPO or IL8) do not behave as independent variables when the hyperferritinemia variable is also present in the analysis. For this reason, in the definitive multivariate analysis, NET markers were excluded and represented with ferritin levels.

One of the major important regulators of NETosis is the DNase I activity, which elicits the crumbling of the traps to recover homeostasis (49). In influenza patients its activity is deficient suggesting that it is a defense mechanism to increase the persistence of NETs and, therefore, their microbicidal activity.

High IL-8 levels and low DNase I activity would facilitate a substantial influx of neutrophils to the lungs, forming long-stable NETs that contribute to infection clearance but also collateral damage to host cells due to the production of toxic molecules for own cells like reactive oxygen species (15). This provokes the destruction of alveolar epithelium and contributes to the development of cytokine storm. In that tendency, the administration of recombinant DNase or biological treatments against the IL-8 axis have reported important benefits in the prognosis of acute respiratory distress syndrome and cystic fibrosis (50, 51).

The analysis of blood levels of ApoH in influenza patients showed that low levels were associated with severe forms, indicating that this molecule behaves as a protective factor for the development of severe forms of the disease, especially those related to RF and hospitalization to ICU. Furthermore, ApoH levels in the entire cohort of influenza patients were significantly lower than those observed in healthy controls. These results are in line with previous publications from our group, where a significant reduction in ApoH levels was observed in COVID-19 and also demonstrated the role of this protein as a protective factor for RF (34). The association of low ApoH levels with severity in infectious disease has been previously described in patients with COVID-19 (34) and sepsis (52). In the same way the presence of ApoH has been identified as a protective factor for organ dysfunction and mortality in sepsis (53). The causes of the reduction of ApoH levels in infections are not well established yet. Among the known facts it stands out that ApoH expression is downregulated both in patients with severe influenza and COVID-19 (54).

ApoH is a key factor in the clearance of apoptotic/necrotic cells avoiding the inherent proinflammatory activity (55). Thus, the consumption of ApoH in the elimination of cellular debris would be added to the effect of the Virus-induced ApoH deregulation causing a situation of partial ApoH deficiency, especially at the local level, which could be related to poor control of proinflammatory cytokines.

The enhancement of the debris removal process has been proposed as a possible therapeutic intervention in inflammatory diseases (56). ApoH replacement therapy in patients with severe influenza and very low levels of the protein may be a suitable alternative. There is currently no possibility of administering recombinant ApoH although fresh plasma (not convalescent) can be used as an indirect way to replenish ApoH with good results in COVID-19 (57, 58). Low levels of ApoH behaved as the independent variable with the greatest statistical strength associated with RF. High levels of IL15 behaved as an independent protective variable. This finding is in contrast to the observation in COVID-19, where elevated levels of IL15 are associated with poor outcome (10), suggesting that the deregulation of the immune response in COVID 19 is different to influenza. Functionally, IL-15 is similar to IL-2 favouring the activation and proliferation of cell-mediated immunity. It may also play a role in the innate immune response to influenza infection (59). If its expression is excessive or chronically deregulated contribute to tissue damage and can be involved in the pathophysiology of the disease (60).

This work presents several limitations. The number of patients recruited is not large enough to confirm in a multivariate analysis subtype-related prognosis differences. Elderly people were considered at risk from a clinical point of view to consider admission to the ICU, although they did not have objective risk criteria. This bias was partially neutralized thanks to the multivariate analysis that allowed the role of each variable to be assessed independently of the age of the individual. The method used for DNase I activity detection exhibits low sensitivity, potentially explaining the lack of differences between severe and non-severe patients. The mRNA expression of ApoH should be assessed in parallel with the quantification of circulating molecule to figure out the reason of the deficiency.

## Data Availability

The raw data supporting the conclusions of this article will be made available by the authors, without undue reservation.
