# Corrigendum: Immune dysregulation is an important factor in the underlying complications in Influenza infection. ApoH, IL-8 and IL-15 as markers of prognosis

**DOI:** 10.3389/fimmu.2024.1472919

**Published:** 2024-08-08

**Authors:** Sara Garcinuño, Antonio Lalueza, Francisco Javier Gil-Etayo, Raquel Diaz-Simón, Ignacio Lizasoain, Ana Moraga, Blanca Diaz-Benito, Laura Naranjo, Oscar Cabrera-Marante, Daniel Enrique Pleguezuelo, Maria Ruiz-Ruigomez, Blanca Ayuso, Estibaliz Arrieta, Dolores Folgueira, Estela Paz-Artal, Cecilia Cueto, Carlos Lumbreras, Antonio Serrano, Manuel Serrano

**Affiliations:** ^1^ Healthcare Research Institute Hospital 12 de Octubre (imas12), Hospital Universitario 12 de Octubre, Madrid, Spain; ^2^ Immunology Department, Hospital Universitario 12 de Octubre, Madrid, Spain; ^3^ Internal Medicine Department, Hospital Universitario 12 de Octubre, Madrid, Spain; ^4^ Faculty of Medicine, Universidad Complutense, Madrid, Spain; ^5^ Red de Infecciones en Inmunodeprimidos no VIH e infecciones relacionadas con la asistencia sanitaria (CIBERINFEC), Instituto de Salud Carlos III, Madrid, Spain; ^6^ Cell Biology Department, Faculty of Medicine, Universidad Complutense, Madrid, Spain; ^7^ Microbiology Department, Hospital Universitario 12 de Octubre, Madrid, Spain; ^8^ Biochemistry Department, Hospital Universitario 12 de Octubre, Madrid, Spain; ^9^ Red de Centros de Investigación Biomédica en Epidemiología y Salud Pública (CIBERESP), Instituto de Salud Carlos III, Madrid, Spain

**Keywords:** influenza, flu, apolipoprotein H, ApoH, β2GPI, IL15, IL8, early immune response

In the published article, there was an error in the **Funding** statement. Incorrect funding bodies and project numbers were listed. The correct **Funding** statement appears below.

“The author(s) declare financial support was received for the research, authorship, and/or publication of this article. This study has been funded by Instituto de Salud Carlos III (ISCIII) (Spanish Ministry of Economy and Competitiveness) through the project “PI20/01361” and the project “PI17/01129” and co-funded by the European Union. And by a grant from Fundación Mutua Madrileña (P177122021).”

The authors apologize for this error and state that this does not change the scientific conclusions of the article in any way. The original article has been updated.

