# Supplementary material for: Immune dysregulation is an important factor in the underlying complications in Influenza infection. ApoH, IL-8 and IL-15 as markers of prognosis
Source: Front Immunol. 2024 Jul 26;15:1443096. doi: 10.3389/fimmu.2024.1443096 (PMC11339618; doi:10.3389/fimmu.2024.1443096)
Supplement: Supplementary file 1 [file Table_1.pdf]

Supplementary Table S1. Cytokines in the reference population of healthy people. The mean values and the standard deviation are indicated, as well as the cut-off point to consider that a sample has high levels of each cytokine.

Cytokines	**Mean**	**Standard Desviation**	**Cut off<br>High levels**
IFN alfa pg/mL	8,1	6.63	>21.4
IFN gamma pg/mL	5.8	6.96	>19.8
IL-2 pg/mL	0.8	1.49	>3.8
IL-6 pg/mL	17.6	23.22	>64
IL-10 pg/mL	6.1	13.62	>3.3
IL-15 pg/mL	1.31	3.14	>7.6
IL-8 pg/mL	6.1	2.9	>11.9
