# Supplementary material for: Immune dysregulation is an important factor in the underlying complications in Influenza infection. ApoH, IL-8 and IL-15 as markers of prognosis
Source: Front Immunol. 2024 Jul 26;15:1443096. doi: 10.3389/fimmu.2024.1443096 (PMC11339618; doi:10.3389/fimmu.2024.1443096)
Supplement: Supplementary file 2 [file Table_2.pdf]

Supplementary Table S2. Characteristics of Influenza patients

CONDITION	Number / median	% / IQR
Sex (women)	112	(54,4%)
Age (years)	75,5	(62-85)
Smoker	21	(10,2%)
Former smoker	52	(25,2%)
Obesity	46	(22,3%)
COPD	43	(20,9%)
Diabetes Mellitus	49	(23,8%)
IFNa elevated	22	(9,1%)
IFNg elevated	19	(9,3%)
IL8 elevated	76	(50,3%)
IL10 elevated	47	(23%)
IL15 elevated	59	(28,9%)
IL2 elevated	8	(3,9%)
IL6 elevated	29	(14,2%)
MPO elevated	47	(32%)
APOH LOW	39	(18,9%)
DNase LOW	182	(88,8%)
CRP elevated	44	(21,6%)
Ferritin elevated	64	(33,5%)
Leucocytosis	60	(29,4%)
Leucopenia	10	(4,9%)
Lymphopenia 1000	133	(65,2%)
Thrombopenia	41	(20%)
Days hospitalized	12,5	(6-14)
Treated in ICU	47	(22,8%)
Respiratory failure	61	(29,6%)
Septic shock	20	(9,7%)
Exitus at 30 days	34	(16,5%)

COPD: chronic obstructive pulmonary disease; MPO: myeloperoxidase; CRP: C-reactive protein; ICU: Intensive Care Unit
