# Supplementary material for: Immune dysregulation is an important factor in the underlying complications in Influenza infection. ApoH, IL-8 and IL-15 as markers of prognosis
Source: Front Immunol. 2024 Jul 26;15:1443096. doi: 10.3389/fimmu.2024.1443096 (PMC11339618; doi:10.3389/fimmu.2024.1443096)
Supplement: Supplementary file 3 [file Table_3.pdf]

Supplementary Table S3. Cytokines. MPO, ApoH and DNase in influenza patients versus reference population of healthy people. The mean values and the standard deviation are indicated, as well as the cut-off point to consider that a sample has high levels of each cytokine.

Biomarker	Reference population		Influenza patients		Hodges-Lehmann median difference	Relative median difference	P value
	Median (IQR)		Median (IQR)				
IFNa pg/mL	7.0	(2.5-12.3)	1.3	(1.1-1.6)	-3.7	-0.49	< 0.001
IFNg pg/mL	4.8	(1.8-7.0)	0.5	(0.4-4.9)	-2.1	-0.43	0.002
IL-8 pg/mL	5.0	(3.5-9.0)	14.8	(3.5-35.8)	8.1	0.55	0.002
IL-10 pg/mL	0.9	(0.9-0.9)	10.8	(1.8-31.7)	7.7	0.71	< 0.001
IL-15 pg/mL	0.2	(0.17-0.17)	2.3	(0.3-7.8)	0.97	0.41	< 0.001
IL-2 pg/mL	0.08	(0.08-1.26)	0.08	(0.02-0.1)	-0.06	-0.75	< 0.001
IL-6 pg/mL	9.0	(0.08-1.26)	9.0	(3.3-41.4)	-0.05	-0.01	0.306
MPO	396	(266-708)	1154	(486-2379)	711	0.62	< 0.001
ApoH (ug/mL)	209	(168-256)	177	(103-281)	-30	-0.14	0.080
DNase (ng/mL)	108	(77-138)	8	(4-8)	-99	-0.93	< 0.001

MPO: myeloperoxidase
