# Supplementary material for: Immune dysregulation is an important factor in the underlying complications in Influenza infection. ApoH, IL-8 and IL-15 as markers of prognosis
Source: Front Immunol. 2024 Jul 26;15:1443096. doi: 10.3389/fimmu.2024.1443096 (PMC11339618; doi:10.3389/fimmu.2024.1443096)
Supplement: Supplementary file 4 [file Table_4.pdf]

Supplementary Table S4. Multivariate analysis of the type of influenza infection and the factors associated with poor disease outcome previously analyzed in the study described in Table 2 B.

	BAD EVOLUTION<br>MULTIVARIATE		
**Biomarker**	**Odds ratio**	**95% CI**	**P value**
Age (years)	0,99	(0,96-1,01)	0,191
Smoker	2,84	(0,83-9,64)	0,095
Obesity	0,47	(0,2-1,09)	0,079
Low ApoH	4,51	(1,73-11,74)	0,002
CRP elevated	2,51	(1,06-5,97)	0,037
Severe lymphopenia	1,82	(0,9-3,71)	0,098
Ferritin elevated	2,35	(1,13-4,87)	0,022
Serotipe influenza A virus	0,68	(0,43-1,07)	0,097
Area under ROC curve	0,794	(0,730-0,850)	

CRP: C Reactive Protein
