# Supplementary material for: Immune dysregulation is an important factor in the underlying complications in Influenza infection. ApoH, IL-8 and IL-15 as markers of prognosis
Source: Front Immunol. 2024 Jul 26;15:1443096. doi: 10.3389/fimmu.2024.1443096 (PMC11339618; doi:10.3389/fimmu.2024.1443096)
Supplement: Supplementary file 5 [file Table_5.pdf]

Supplementary Table S5. Multivariate analysis of the markers associated with respiratory failure detected in the univariate analysis of the. 61 influenza patients who followed this evolution versus those who maintained adequate ventilation.

	Respiratory failure UNIVARIATE		Respiratory failure MULTIVARIATE		
Biomarker	Odds ratio	95% CI	Odds ratio	95% CI	P value
Age (years)	0.98	(0.96-0.99)	0.98	(0.96-1.01)	0.158
Smoker	5.87	(2.24-15.43)	7.32	(1.22-44.06)	0.030
COPD	2.59	(1.29-5.19)	3.16	(1.05-9.51)	0.041
IL8 elevated	2.69	(1.28-5.65)	1.63	(0.67-3.93)	0.280
IL10 elevated	2.36	(1.2-4.65)	2.39	(0.92-6.2)	0.072
IL15 elevated	0.44	(0.21-0.91)	0.30	(0.1-0.89)	0.029
Low ApoH	4.25	(2.05-8.79)	4.40	(1.51-12.84)	0.007
CRP elevated	2.42	(1.21-4.83)	2.06	(0.76-5.58)	0.157
Area under ROC curve			0.792	(0.718-0.854)	

COPD: chronic obstructive pulmonary disease; CRP: C-reactive protein.
